# Supplementary material for: Hierarchical triphase diffusion photoelectrodes for photoelectrochemical gas/liquid flow conversion
Source: Nat Commun. 2023 May 8;14:2643. doi: 10.1038/s41467-023-38138-9 (PMC10167308; doi:10.1038/s41467-023-38138-9)
Supplement: Supplementary file 1 — Supplementary Information [file 41467_2023_38138_MOESM1_ESM.pdf]

Supplementary Information

Hierarchical triphase diffusion photoelectrodes for photoelectrochemical gas/liquid flow conversion

Xiangyu Meng[1,6], Chuntong Zhu[1,6], Xin Wang[2,6], Zehua Liu[3], Mengmeng Zhu[1], Kuibo Yin[4], Ran Long[3], Liuning Gu[5], Xinxing Shao[5], Litao Sun[4], Yueming Sun[1], Yunqian Dai[1,]* & Yujie Xiong[2,3]*

[1]School of Chemistry and Chemical Engineering, Southeast University, Nanjing, Jiangsu 211189, China.

[2]Anhui Engineering Research Center of Carbon Neutrality, School of Chemistry and Materials Science, Anhui Normal University, Wuhu, Anhui 241000, China.

[3]School of Chemistry and Materials Science, Hefei National Laboratory for Physical Sciences at the Microscale, and National Synchrotron Radiation Laboratory, University of Science and Technology of China, Hefei, Anhui 230026, China.

[4]School of Electronic Science and Engineering, Southeast University, Nanjing, Jiangsu 211189, China.

[5]School of Civil Engineering, Southeast University, Nanjing, Jiangsu 211189, China.

[6]These authors contributed equally: Xiangyu Meng, Chuntong Zhu and Xin Wang.

* Corresponding author.

E-mail: yjxiong@ustc.edu.cn; daiy@seu.edu.cn

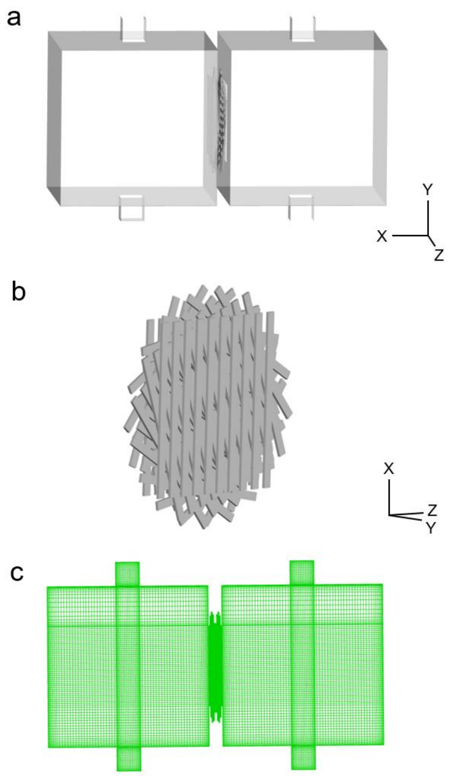

Supplementary Fig. 1 Fluid simulation model of (**a**) flow cell and (**b**) interwoven fibrous channels. (**c**) Mesh generation in fluid simulation.

Notably, the constructed models in (b) were the channels for fluid transfer.

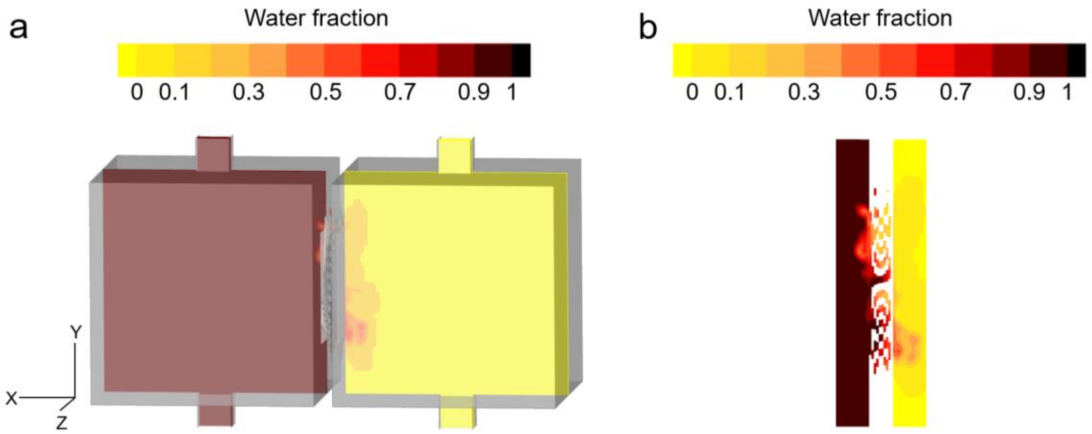

Supplementary Fig. 2 The fluid simulation showing the water fraction (**a**) in reactor and (**b**) on pore channels, respectively. The color bars represent water fractions.

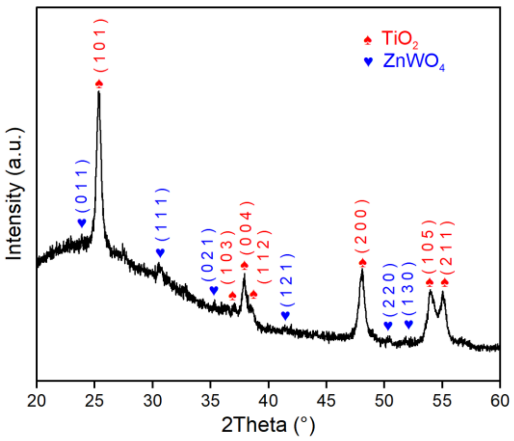

Supplementary Fig. 3 XRD pattern of $TiO_2/ZnWO_4$ fibrous mat.

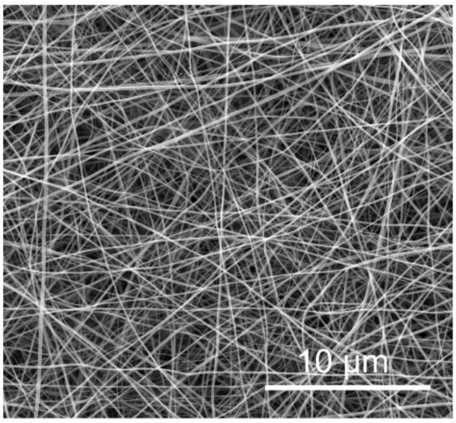

Supplementary Fig. 4 SEM image of R-TiO_2/$ZnWO_4$ fibrous mat.

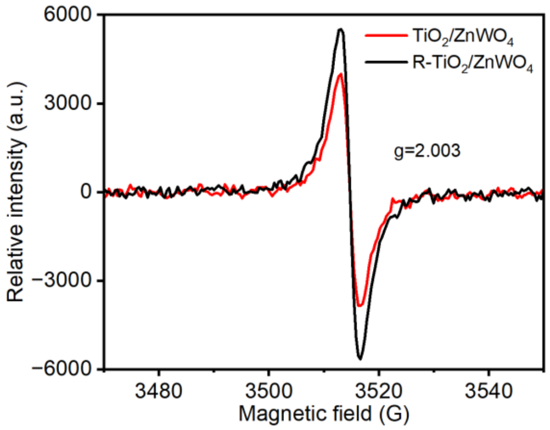

Supplementary Fig. 5 EPR spectra of $TiO_2/ZnWO_4$ and R-$TiO_2/ZnWO_4$ fibrous mats in dark with the same sample weight.

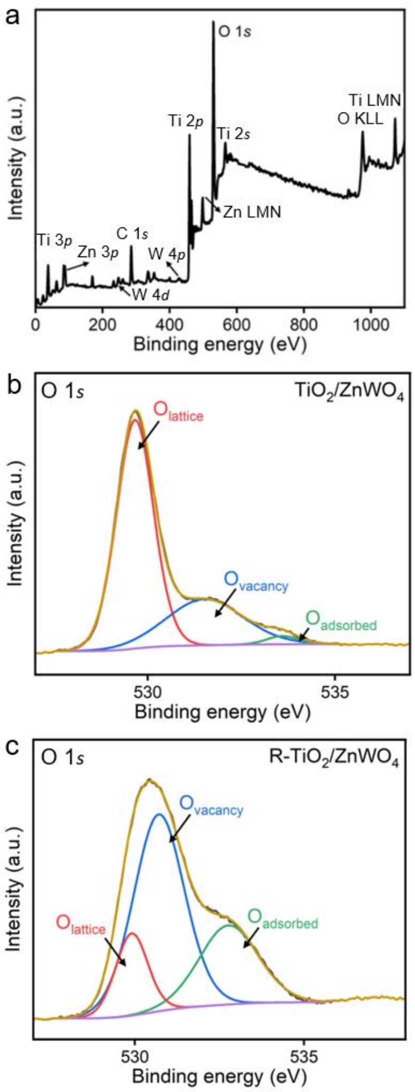

Supplementary Fig. 6 (**a**) XPS survey spectrum of R-TiO_2/$ZnWO_4$ fibrous mat, and high-resolution O 1*s* XPS spectra of (**b**) TiO_2/$ZnWO_4$ and (**c**) R-TiO_2/$ZnWO_4$ fibrous mats.

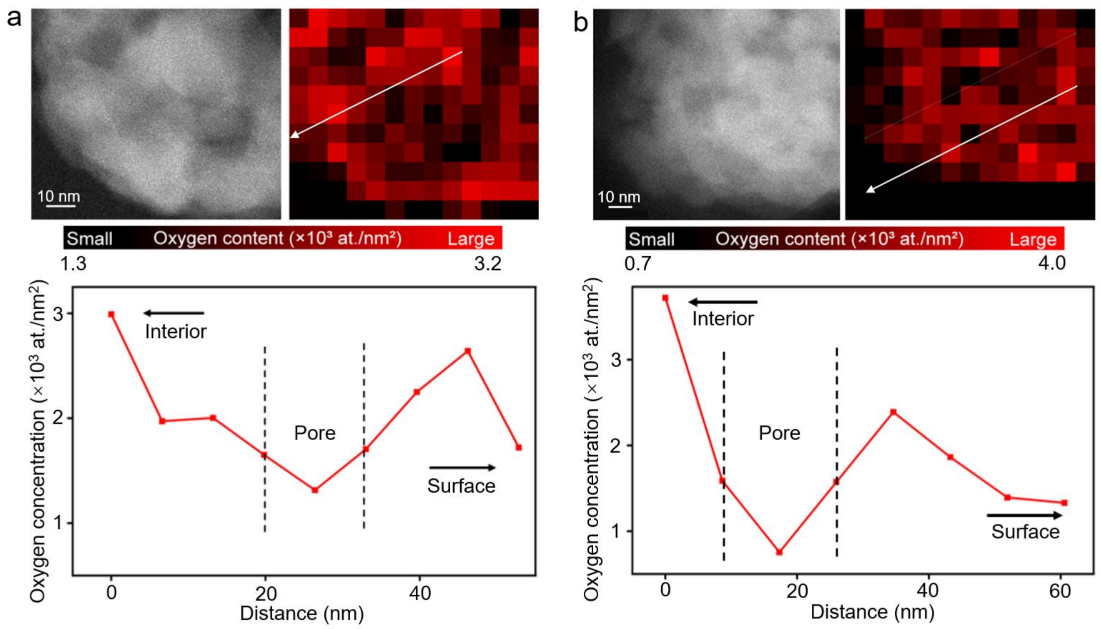

Supplementary Fig. 7 STEM image and corresponding EELS mapping profiles of oxygen atoms for two different nanofiber FIB slices. The analysis results of oxygen content distribution on a nanofiber slice are represented by the data graphs. The white arrows highlight the scanning routes in mapping. The color bar in left represents the oxygen content ($\times 10^3$ at./nm^2) on slice.

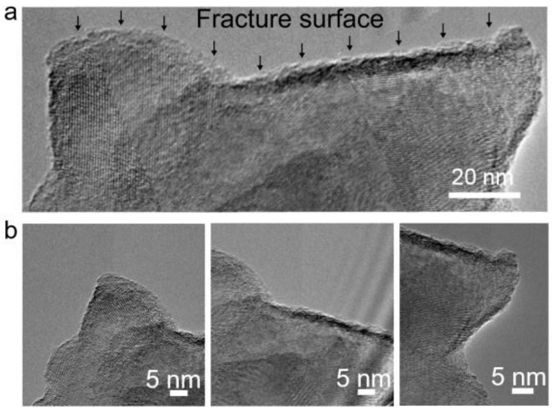

Supplementary Fig. 8 (**a**) TEM image showing the fracture surface of nanofiber after mechanical test under *in-situ* TEM observation. The image in (**a**) was patched by the TEM images in (**b**).

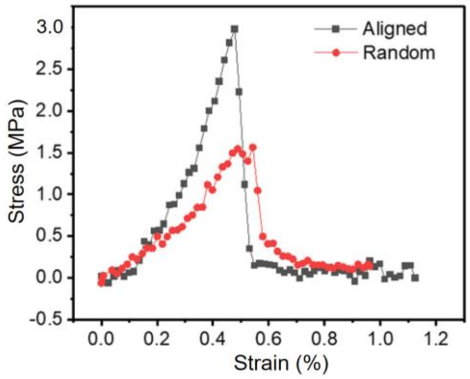

Supplementary Fig. 9 Mechanical tensile test of $TiO_2/ZnWO_4$ fibrous mats with aligned and random assembly.

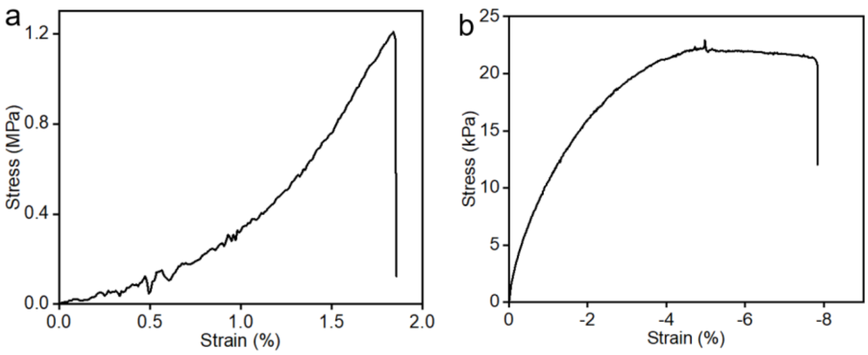

Supplementary Fig. 10 Mechanical (**a**) tensile and (**b**) compression test of R-TiO_2/$ZnWO_4$ fibrous mats.

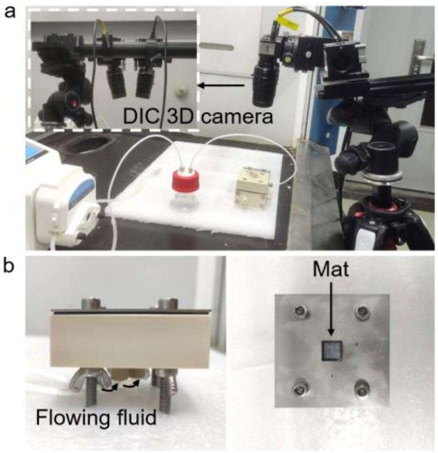

Supplementary Fig. 11 Photographs of (**a**) DIC 3D camera and (**b**) a flow reactor with mat for the DIC test setup. The fluid flow rate was controlled by peristaltic pump.

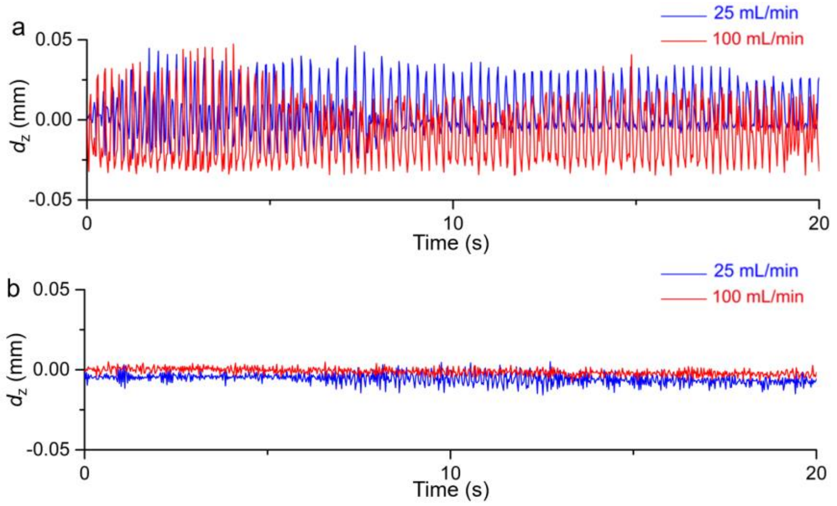

Supplementary Fig. 12 DIC test for observation micro-strain within mat by flowing (**a**) electrolyte and (**b**) CH_4 fluid with flow rates of 25 and 100 mL/min, respectively.

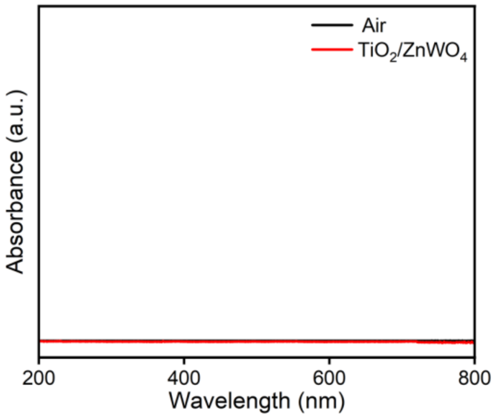

Supplementary Fig. 13 UV-vis absorption spectra of the transparent $TiO_2/ZnWO_4$ fibrous mat.

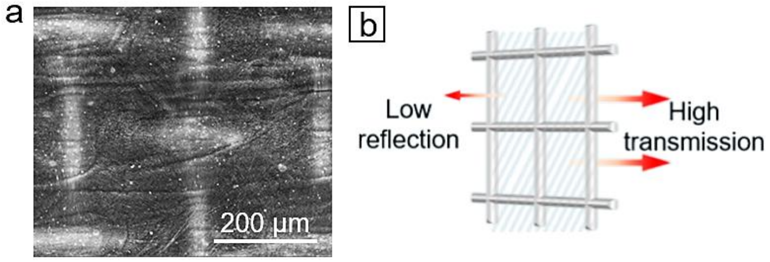

Supplementary Fig. 14 (**a**) SEM image showing the microstructures of transparent mat, and (**b**) schematic functional illustration.

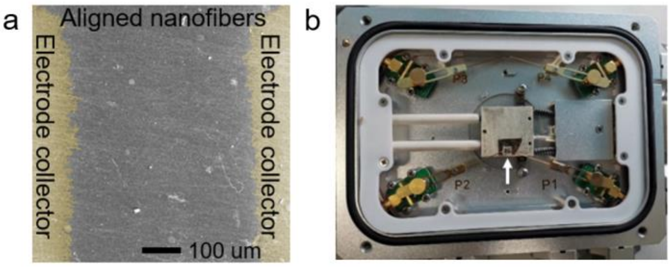

Supplementary Fig. 15 (**a**) SEM image showing the conductivity test chip design, including Au electrode collectors (highlighted by gold color) and aligned nanofibers. (**b**) The setup with probes for testing current–voltage curves. The white arrow highlights the location of fibrous chip.

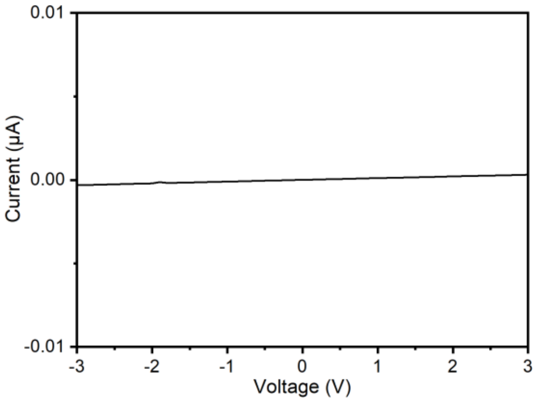

Supplementary Fig. 16 The inherent conductivity test of $TiO_2/ZnWO_4$ with interwoven fibrous structure.

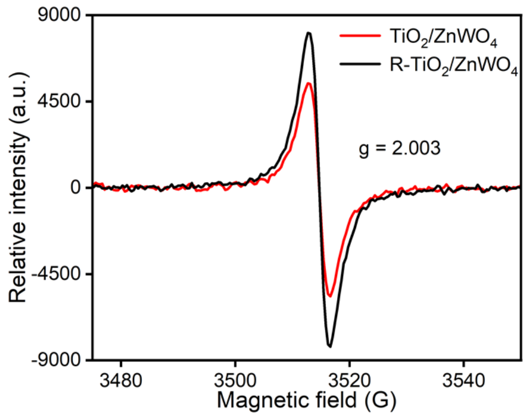

Supplementary Fig. 17 EPR spectra of $TiO_2/ZnWO_4$ and R-$TiO_2/ZnWO_4$ fibrous mat under light irradiation with the light intensity of 1 Sun and the same sample weight.

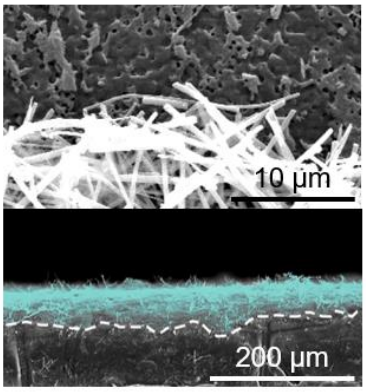

Supplementary Fig. 18 SEM images showing the R-TiO_2/$ZnWO_4$/PTFE fibrous mat with pores on both R-TiO_2/$ZnWO_4$ and PTFE from a top view (top), and their closely combined interface from a cross-sectional view (bottom).

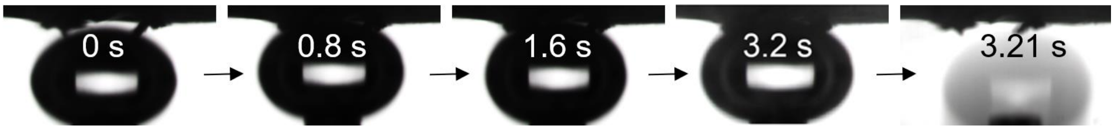

Supplementary Fig. 19 The contact angles of CH_4 gas bubbles on the surface of R-TiO_2/$ZnWO_4$ fibrous mat in the $NaSO_4$ (pH = 2) liquid.

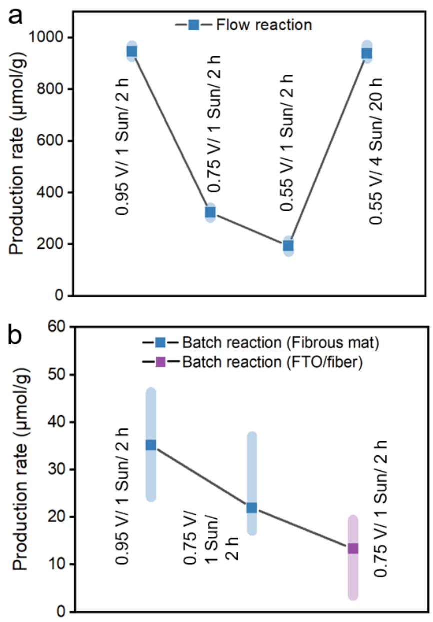

Supplementary Fig. 20 The production of CO_2 in PEC CH_4 conversion (**a**) in flow reaction and (**b**) in batch reaction. The error bars represent standard deviations in (**a**) and (**b**).

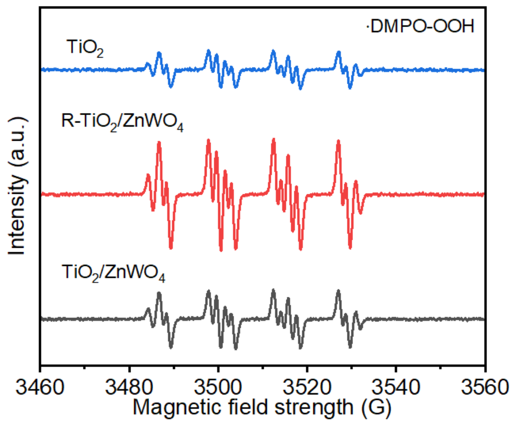

Supplementary Fig. 21 EPR spectra of samples under light irradiation (1 Sun) and in electrolyte (Na_2SO_4, pH = 2) for simulating the reaction conditions.

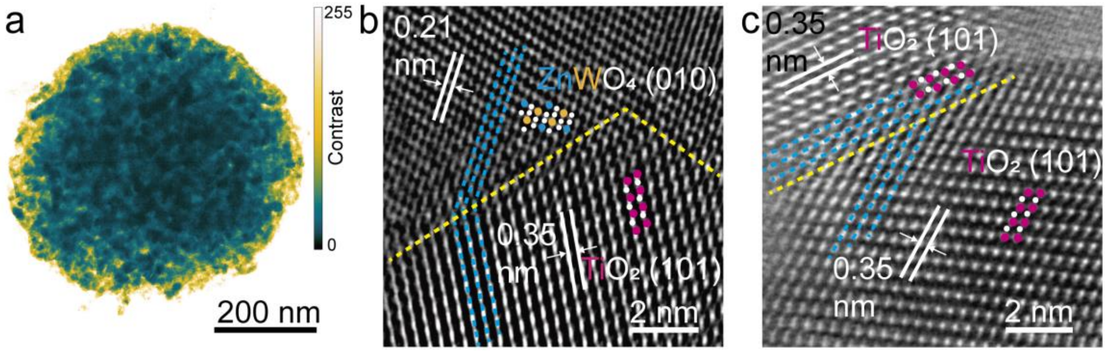

Supplementary Fig. 22 (**a**) TEM image showing the $TiO_2/ZnWO_4$ counterpart sample prepared through electrospray. The black-white color map was changed into a green-yellow color map by Digital Morphology software to better show the grains inside the thick sample, with the inserted color bars for representing the contrast. (**b**, **c**) HRTEM images of grain boundaries within the counterpart sample. In (**b**) and (**c**), Ti, O, Zn and W atoms are highlighted by magenta red, white, blue and orange dots, respectively; ordered and continuous crystal lattices are marked with blue dashed lines; grain boundaries are highlighted by yellow dashed lines; lattice spacing is highlighted by white lines in pairs.

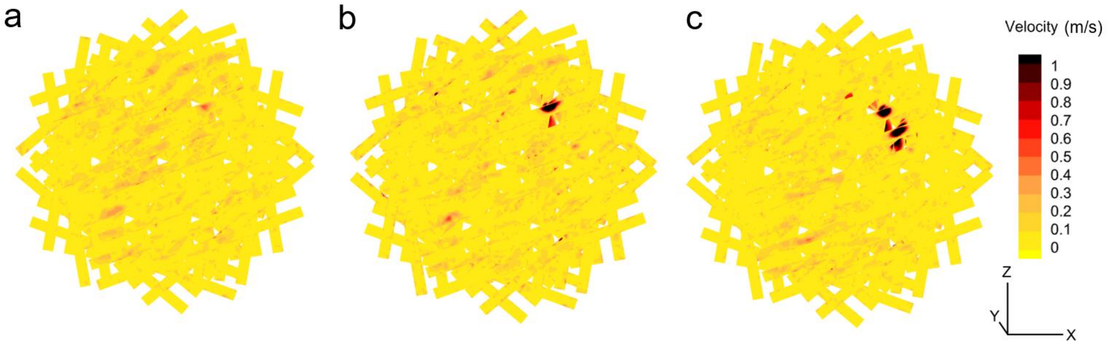

Supplementary Fig. 23 Fluid simulation showing the diffusion rates of CH_4 gas on micropores among interwoven nanofibers, with the increase of the flow rate of CH_4 gas from (**a**) 25 mL/min to (**b**) 50 mL/min and (**c**) 100 mL/min. The color bar represents the velocity of mass diffusion.

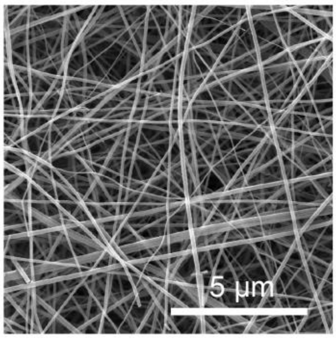

Supplementary Fig. 24 SEM image showing the intact fibrous mat after 20 h stability test.

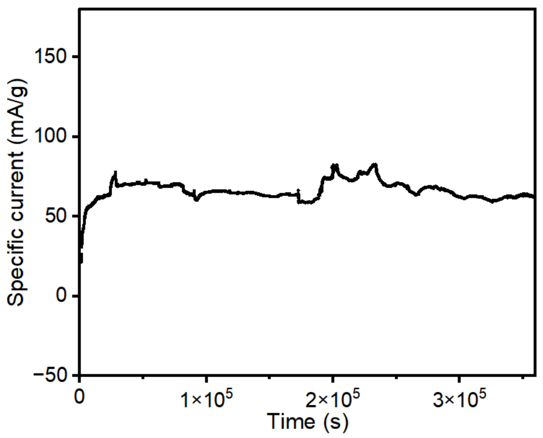

Supplementary Fig. 25 Specific current of CH_4 conversion in flow cell by diffusion fibrous mat at 0.55 V under 1 Sun light irradiation for 100 h running time.

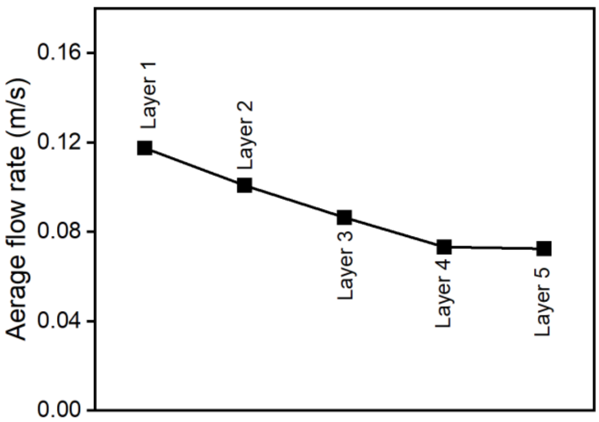

Supplementary Fig. 26 Relationship between the fibrous mat layer numbers and the average fluid flow rates in simulations.

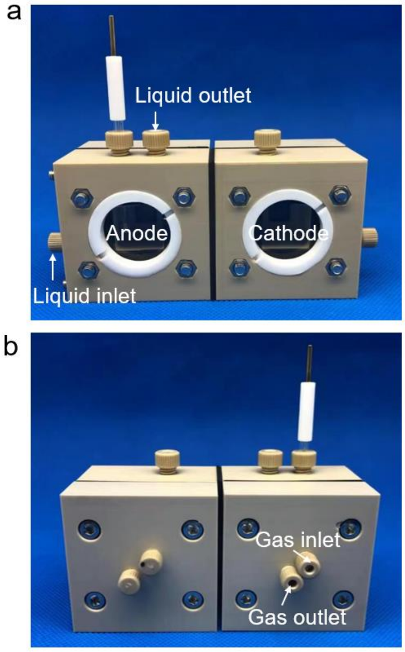

Supplementary Fig. 27 Optical images showing the flow reactor from (**a**) light irradiation side and (**b**) back view.

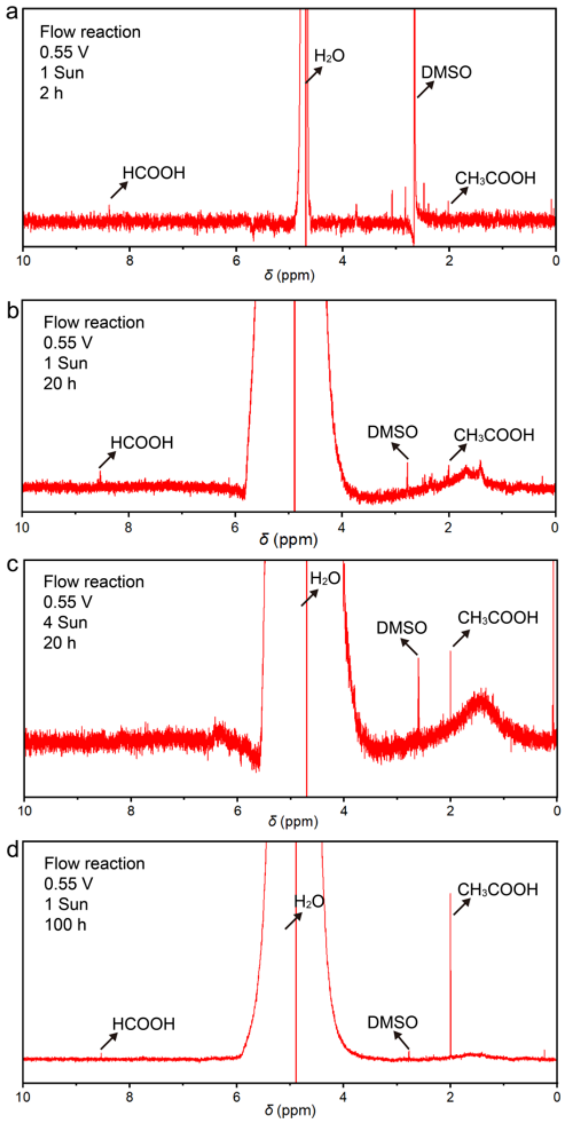

Supplementary Fig. 28 The ^{1}H NMR spectra of liquid products in different flow reaction conditions.

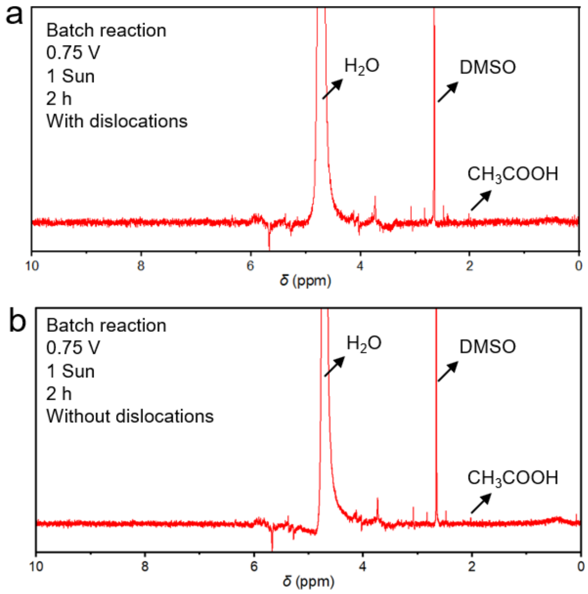

Supplementary Fig. 29 The ^{1}H NMR spectra of liquid products in batch reaction conditions by the R-TiO_2/$ZnWO_4$ samples (**a**) with and (**b**) without dislocations.

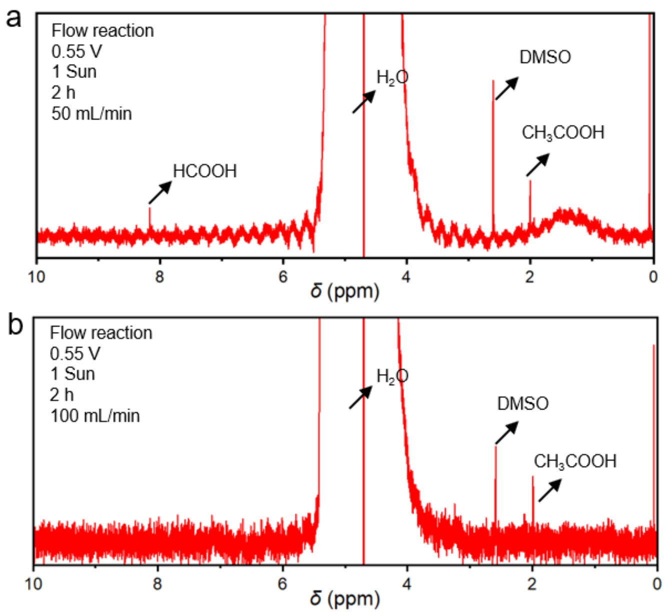

Supplementary Fig. 30 The ^{1}H NMR spectra of liquid products in flow reactions by the R-TiO_2/$ZnWO_4$ mat with CH_4 flow rates of (**a**) 50 mL/min and (**b**) 100 mL/min.
