# Supplementary material for: Hierarchical triphase diffusion photoelectrodes for photoelectrochemical gas/liquid flow conversion
Source: Nat Commun. 2023 May 8;14:2643. doi: 10.1038/s41467-023-38138-9 (PMC10167308; doi:10.1038/s41467-023-38138-9)
Supplement: Supplementary file 3 — Description of Additional Supplementary Files [file 41467_2023_38138_MOESM3_ESM.pdf]

Description of Additional Supplementary Files

File Name: Supplementary Movie 1
Description: 5,000-cycling bending test of TiO_2/$ZnWO_4$ fibrous mat.
